# Editorial: Hypoxia in Kidney Disease

**DOI:** 10.3389/fphys.2018.00485

**Published:** 2018-05-03

**Authors:** Fredrik Palm, Maarten P. Koeners

**Affiliations:** ^1^Department of Medical Cell Biology, Uppsala University, Uppsala, Sweden; ^2^Institute of Biomedical and Clinical Science, University of Exeter Medical School, University of Exeter, Exeter, United Kingdom; ^3^School of Physiology, Pharmacology and Neuroscience, Biomedical Sciences, University of Bristol, Bristol, United Kingdom

**Keywords:** kidney hypoxia, chronic kidney disease, hypertension, magnetic resonance imaging, mitochondrial uncoupling, telemetry, kidney transplantation, sympathetic nerve activity

## Introduction

Oxygen was first described by Carl Wilhelm Scheele as “Fire air” since it supported combustion. He obtained oxygen by heating mercuric oxide, silver carbonate, and nitrate salts. Scheele communicated his findings to Lavoisier, who realized the significance of this finding. Scheele's discovery of oxygen (ca. 1771) was chronologically earlier than the corresponding work of Priestley and Lavoisier, but he did not publish this discovery until 1777, after both of his rivals had already published their findings (West, 2014). Because others generally are accredited for the discovery of oxygen, and a number of other discoveries, he was nicknamed “hard-luck Scheele.”

Oxygen is essential for aerobic metabolism, a fundamental mechanism for energy production. The delivery of optimal levels of oxygen to tissues is tightly regulated as both hypoxia and hyperoxia are detrimental for cellular function. Indeed, tissue hypoxia has been found during pathological conditions such as cancer (Liu et al., 2016), diabetes (Palm et al., 2003), hypertension (Welch et al., 2001), chronic kidney disease (CKD) (Milani et al., 2016), and stroke (Ferdinand and Roffe, 2016). In the 90's Fine et al. proposed kidney hypoxia as a mediator of progressive kidney disease (Fine et al., 1998). Since then, experimental and clinical studies have solidified the view that kidney hypoxia plays a critical role during the genesis and progression of both acute and CKD. This research field is currently at the beginning of integrating pre-clinical with clinical research in which kidney hypoxia related mechanisms are quantified by non-invasive imaging. In combination with the fact that some key questions remain unanswered, this offers exciting new research perspectives that are waiting to be explored. With this Frontiers Research Topic we discuss and identify potential mediators/controllers of hypoxia in kidney disease. If we understand more about the sequence of events leading to kidney hypoxia, its regulation and consequences in renal disease, we might be able to have a major impact in clinical practice. I.e., more accurate and earlier diagnosis, novel treatment targets, and novel therapies.

## Hypoxia in kidney disease

Liu et al. describes the alterations in renal oxygenation (delivery, consumption and tissue oxygen tension) in pre-clinical and clinical studies in diabetic and hypertensive CKD along with the underlying mechanisms and potential therapeutic options. The novel hypothesis by Patinha et al. on the cooperative oxygen sensing by the kidney and carotid body in blood pressure control, could further explain the role of kidney hypoxia in hypertension. This is relevant for the pursuit of novel ways to treat associated with sympathetic overdrive. Indeed, Hering et al. reduced sympathetic nerve activity and lowered blood pressure in CKD patients using 100% oxygen (Hering et al., 2007). Furthermore, van der Bel et al. assessed the underlying hemodynamic modulation and found that oxygen supplementation in CKD patients caused a non-baroreflex-mediated increase in vascular resistance and blood pressure. Conceivably, within their experimental set-up, the effects of oxygen on systemic vasoconstriction (leading to baroreflex deactivation with reduction in sympathetic tone) did not allow for the detection of any possible subtle effects of kidney specific oxygenation on sympathetic outflow.

Maekawa et al. describes that elevated levels of the uremic toxin indoxyl sulfate (IS) in CKD can induce endoplasmatic reticulum (ER) stress and subsequent hypoxia through suppression of erythropoiesis and exacerbation of tubular fibrosis. They propose that the removal of IS, blockage of aryl hydrocarbon receptor (mediator of IS-induced suppression of erythropoietin production), inhibition of hepcidin production (controls iron homeostasis) and mediation of the ER unfolded protein pathway could be therapeutic targets in ameliorating hypoxia and subsequent CKD progression. Preserving mitochondrial uncoupling might be a potential treatment target. Indeed, kidney hypoxia *per se*, caused by mitochondrial uncoupling, without confounding factors such as uraemia, hyperglycaemia or hypertension results in albuminuria and tubulointerstitial damage (Friederich-Persson et al., 2013). Schiffer et al. proposes that diabetic nephropathy is likely a joint mechanism of mitochondrial reactive oxygen species production, which include altered mitophagy, mitochondrial dynamics, mitochondrial uncoupling, and signaling through AMP-activated protein kinase and hypoxia-inducible factors. Therefore, prevention or correction of mitochondrial dysfunction might be pivotal in treating diabetic kidney disease.

## Hypoxia quantification in kidney disease

Hirakawa et al. addresses the pathophysiological importance of renal hypoxia with a focus on hypoxia detection and quantification. Although kidney tissue oxygenation has been studied for a long time, recent advances have made it possible to achieve continuous measurements, non-invasive oxygen assessments, and intracellular oxygen assessments. Using oxygen tension telemetry Emans et al. demonstrate circadian variation in kidney tissue oxygenation with maximal values during the lights-off period, when renal excretion of electrolytes was highest. These type of experiments will allow long-term investigation of kidney oxygenation in relation to disease development, including the investigation of circadian influence on normal physiology and disease development (Adamovich et al., 2017). Blood Oxygenation-Level Dependent (BOLD) magnetic resonance imaging (MRI) allows for non-invasive estimation of kidney oxygenation in humans, without the need for administration of exogenous contrast agents. Pruijm et al. summarizes the growing knowledge of factors that influence the BOLD-signal and how this has led to better standardization, refinements, and reproducibility. BOLD, alone or in combination with other MRI modalities, could therefore contribute to patient stratification, earlier diagnosis, and improve long-term follow up. Indeed, Eijs et al. report that functional MRI (including BOLD) has the potential to detect several pathophysiological mechanisms involved in kidney allograft dysfunction, making it a promising tool in long-term follow-up of kidney transplantation patients. In addition, Cox et al. showed in a pilot study that multiparametric MRI could assess renal structure, hemodynamics and oxygenation in healthy participants and CKD patients. Renal blood flow and renal cortex perfusion was lower in CKD patients compared with healthy participants. Longitudinal relaxation time (T_1_ values), an MRI parameter that increases with reduced perfusion/filtration and/or progressive scarring, were increased in both renal cortex and medulla compared to healthy participants, though primarily in cortex, resulting in a loss of corticomedullary differentiation. Finally, Laustsen describes how the introduction of dissolution dynamic nuclear polarization technology has enabled a new paradigm for renal MRI. They describe the utility of hyperpolarized MRI in preclinical research in which the real-time interrogation of metabolic turnover has already aided the physiological and pathophysiological metabolic and functional effects in *ex vivo* and *in vivo* models, and discus its potential translation to renal patients.

## Conclusion

This research topic highlights the important role of hypoxia in kidney disease and what progress has been made to address its importance. We conclude that it is imperative to further advance our understanding about the sequence of events leading to intrarenal tissue hypoxia, its regulation and consequences in both acute and CKD. If we do that, we might be able to end Scheele's “hard-luck” and have a major impact of patient care and reduce the poor quality of life often associated with kidney disease.

## Author contributions

MK: conceived the content and drafted the manuscript; MK and FP: revised and approved the final manuscript.

### Conflict of interest statement

The authors declare that the research was conducted in the absence of any commercial or financial relationships that could be construed as a potential conflict of interest.
